# Extracellular vesicles derived from M1 macrophages enhance rat midpalatal suture expansion by promoting initial bone turnover and inflammation

**DOI:** 10.1186/s40510-023-00477-0

**Published:** 2023-09-04

**Authors:** Yi Liu, Yuan Zhong, Bowen Zheng, Yi Liu

**Affiliations:** Department of Orthodontics, School and Hospital of Stomatology, Liaoning Provincial Key Laboratory of Oral Disease, China Medical University, Shenyang, 110002 China

**Keywords:** Suture expansion, Midpalatal suture, Rapid maxillary expansion, Injection, Extracellular vesicles

## Abstract

**Background:**

Midpalatal suture (MPS) expansion can be affected by many factors, and researchers have attempted to regulate the initial inflammatory stage of expansion to optimize clinical outcomes and their underlying mechanisms. This study aimed to investigate the potential effects and mechanisms of M1 macrophage small extracellular vesicles during rat MPS expansion.

**Materials and methods:**

RAW264.7 cells were induced to M1 or M2 polarization and, small extracellular vesicles were isolated from the polarized macrophages. Male Sprague–Dawley rats (6–7 weeks) were administered 70 ± 5 g expansion force devices for 7 days. Rats with expanders without force served as controls. M1/M2 small extracellular vesicles were injected into the MPS region (50 µg/day) in the M1 and M2 small extracellular vesicle-assisted groups, while 0.9% saline was injected into the expansion-only group. Suture width, bone mass, and morphological changes in the region of interest (ROI) were examined.

**Results:**

The M1 small extracellular vesicle-assisted group showed a significantly increased MPS suture width in vivo (P < 0.001), and less bone mass was observed in the ROI (P < 0.05). Histological examination showed that the M1 small extracellular vesicle-assisted group exhibited a wider palatal area and obvious fibrous tissue rearrangement. The expression of RANKL and the number of osteoclasts were increased (P < 0.01) in the bony edges, and the p65 protein expression was significantly higher (P < 0.001).

**Conclusions:**

M1 macrophage-derived small extracellular vesicles have a positive effect in MPS expansion and increase p65 protein content and RANKL expression, thus promoting bone turnover. This study may contribute to the clinical application of small extracellular vesicles in the expansion of the palatal suture.

## Introduction

Midpalatal suture (MPS) expansion is a preferred orthodontic therapy for midfacial hypoplasia, such as crowding, crossbite, maxillary transverse skeletal deficiency, and V-shaped upper dental arch [1, 2]. The expansion stresses are conducted from molars and the alveolar bone to the suture, resulting in skeletal remodeling and finally reaching a new equilibrium [3]. This process is affected by cell signaling pathways as well as cytokines and transcription factors. Inflammation and osteoclast resorption at the initial stage following force application are required for MPS expansion [4], and the rate of bone turnover is the key to clinical effectiveness [5]. However, few studies have concentrated on the specific early stage of MPS expansion and subsequent bone remodeling.

The process of expansion is marked by initial macrophages flooding the dilation site [6]. Macrophages are critical regulators in innate immunity and are involved in bone tissue metabolism [7]. The reduction in macrophages has a significant impact on cartilage ossification in a mouse model of femoral fracture [11]. Resting macrophages can be polarized into the classically activated (M1) type and the alternatively activated (M2) type. These cells have different functional plasticities by secreting various cytokines, chemokines, and other bioactive factors, such as extracellular vesicles (EVs), to modulate the local microenvironment [12]. Recent research has focused on affecting bone remodeling using nonsurgical procedures [13]. The effect of MPS expansion in rats was diminished when bisphosphonates suppressed osteoclasts and the initial inflammatory response [4]. Thus, modulating macrophage-associated inflammation may improve expansion outcomes and have an impact on later bone remodeling.

Extracellular vesicles (EVs) are lipid bilayer-enclosed vesicles shed from almost all cell types containing proteins, metabolites, nucleic acids, and lipids of the parental cells [14]. These vesicles are classified mainly into exosomes, microvesicles, and microparticles by specific mechanisms. Due to the inability of current separation techniques to precisely separate exosomes and microvesicles in size and density, we refer to EVs (< 100 nm or < 200 nm) precipitated at high speeds (above 100,000 g) as small-sized EVs (sEVs) according to the updated guidelines of the International Society for Extracellular Vesicles of 2018 (MISEV2018) [14]. M1/M2 macrophage EVs can mimic the function of their parental cells, which have diverse roles in osteogenesis and may transiently mediate inflammatory pathways[15]. M1 macrophage EVs have been shown to enhance the transcriptional activation of the inflammatory pathway NF-κB, as well as immunological inflammatory responses [16]. M2 macrophage EVs, in contrast, decrease immunological responses and aid bone tissue regeneration [17]. Kang [18] confirmed that M1 EVs containing miR-155 negatively affected osteogenesis in vivo, while M2 EVs containing miR-378a had a positive effect on osteoinduction, suggesting that macrophage EVs can promote immunomodulation during bone remodeling.

Investigations have been performed to increase the therapeutic effectiveness of orthodontic therapy and shorten the treatment time [4, 19]. Early bone resorption is followed by bone apposition. This experiment evaluated the clinical efficiency of M1 macrophage sEVs (M1-sEVs) in a rat MPS expansion model and further revealed the functional mechanism. We hypothesized that M1 macrophage sEVs could enhance the inflammatory response during the early stages and regulate bone metabolism. We discovered that locally applying an M1-sEV suspension might increase the in vivo expression of expansion force and the efficiency is supported by a potential NF-κB proinflammatory pathway.

## Materials and methods

### Cell culture

RAW264.7 cells (RAW Mϕs, Stem Cell Bank, Chinese Academy of Sciences) between Passage 2-Passage 25 were used. The cells were cultured in 100-mm culture dishes at a density of 1 × 10^6^ supplemented with high glucose Dulbecco’s Modified Eagle’s Medium (DMEM; Gibco, USA), 10% fetal bovine serum (FBS; Clark, USA), 0.292 mg/ml L-glutamine (Invitrogen, USA), and 1% penicillin and streptomycin (Hyclone, USA) at 37℃ with 5%CO_2_. The cells were treated with 80 ng/ml interferon γ (IFN-γ; peprotech, USA) or 20 ng/ml IL-4 (peprotech, USA) for 24 h respectively to stimulate M1 or M2 phenotype after reaching a contact rate of roughly 60%. Although IFN-γ or lipopolysaccharide (LPS) was included in the classical activation into M1 phenotype [20], LPS could reside in exosomes and affect animal trials. We utilized IFN-γ alone in M1 activation [21]. M2 macrophages served as a control since they are another subtype of polarized macrophages. Cells supplemented with phosphate-buffered saline (PBS) are considered M0 macrophages. Before performing any cell experiments, a hemocytometer was used to determine cell density.

### Identification of M1, M2 polarized macrophages

Flow cytometry analysis was used to identify the M1 macrophage immunophenotype after 24 h of induction. 1 × 10^6^ macrophages were scraped with cell scrapers (Greiner bio-one, Germany) and washed twice in PBS after being treated with PBS, IFN, or IL-4. The cells without antibody served as the blank control, while the other treatment groups were incubated on ice for 30 min in the dark with APC anti-mouse CD86 antibody (Biolegend, USA) before being resuspended in 450ul PBS containing 2% FBS. The analysis was conducted with BD FACSCanto II cytometer(BD, USA), and the data were analyzed using FlowJo software.

The quantitative real-time polymerase chain reactions (qRT-PCR) were performed with TB Green Premix EX Taq™ II (Takara, Tokyo, Japan) following the manufactures’ instructions. The mRNA expression levels of Inducible nitric oxide synthase (iNOS), Interleukin-1 β (IL-1β), Arginine-1 (Arg1), and Transforming growth factor-β (TGF-β) were identified. TRizol reagent (Takara, Tokyo, Japan) was used for total RNA extraction. A Prism 7500 real-time PCR apparatus was used to perform PCR-specific amplification (AB Quantstudio3, Thermo Fisher, USA), and the fold change of gene expression was analyzed with the 2^−△△CT^ method after normalizing all expression data to the housekeeping gene GAPDH. Primer sequences are shown in Table 1.

**Table 1:** Primer sequences for quantitative real-time polymerase chain reaction (qRT-PCR) analysis

Genes	Full name	Forward (5′–3′)	Reverse (5′–3′)
iNOS	Inducible nitric oxide synthase	ATCTTGGAGCGAGTTGTGGATTGTC	TAGGTGAGGGCTTGGCTGAGTG
IL-1β	Interleukin1-β	CACTACAGGCTCCGAGATGAACAAC	TGTCGTTGCTTGGTTCTCCTTGTAC
Arg-1	Arginine-1	CATATCTGCCAAAGACATCGTG	GACATCAAAGCTCAGGTGAATC
TGF-β	Transforming growth factor-β	GCAACAATTCCTGGCGTTACCTTG	CAGCCACTGCCGTACAACTCC
GAPDH	GAPDH	GTTGTCTCCTGCGACTTCA	TGGTCCAGGGTTTCTTACTCC

### Isolation and characterization of M1, M2 macrophage sEVs

sEVs for animal experiments were collected via differential ultracentrifugation. FBS was centrifuged at 130,000 × g for 18 h using an ultracentrifuge(Beckman Optima XPN-100, 32Ti ultra rotor, USA) and passed through 0.22-μm pore filters (Millipore, USA) as sEV-depleted FBS [23]. The original medium of M1 and M2 macrophages was discarded and the cells were washed twice with PBS, then incubated in DMEM supplemented with 10% sEV-depleted FBS for 24 h separately. The culture medium was centrifuged at 300 g for 4 min, 3000 g for 15 min, 10000 g for 30 min, filtered on 0.22-μm pore filters in order (Thermo, USA) to remove dead cells, living cells, cell debris, and then centrifuged at 130,000 g for 120 min to obtain the pellet (sEVs). All procedures were carried out at 4 ℃. The sEVs were resuspended in sterile PBS and quantified with a Bicinchoninic Acid assay kit (BCA; Meilune, China). M1, and M2-sEVs were stored at -80 °C after being diluted to 1 μg/ul before use.

To observe the sEVs morphology, 10 μl of fresh sEVs solution was fixed with 2.5% glutaraldehyde and dropped onto the copper mesh, adsorbed at room temperature for about 5 min. The sEVs were then stained for 1 min at room temperature using 10 μl of saturated uranyl acetate solution dropwise applied to the copper grid. The samples were examined with an 80 kV transmission electron microscope (TEM; JEOL JEM-1230, Japan). The membrane markers of sEVs were verified by western blotting. The protein concentration was measured using a BCA assay kit and proteins were lysed with RIPA buffer (Thermo, USA) and maintained on ice. Protein samples (40ul) were combined in a 5:1 ratio with loading buffer (6 × SDS) and placed onto a 10% sodium dodecyl sulfate polyacrylamide gel electrophoresis. Proteins were transferred to 0.22um polyvinylidene fluoride membranes after electrophoresis and blocked for 3 h. Then membranes were probed with CD9 (1:1000, Thermo, USA), CD63 (1:1000, Thermo, USA), and Alix (1:1000, Thermo, USA) primary antibodies overnight at 4 °C according to the antibody specifications. Next, membranes were washed three times with 5% Bovine Serum Albumin (BSA) for 5 min each time, then incubated for 1 h at room temperature before being treated with horseradish peroxidase-labeled goat anti rabbit IgG (HRP; 1:3000, Abcam, UK). The 4200 SF chemiluminescence detection device was used to visualize the signals (Tanon, China). The particle number and size of M1, M2-sEVs were detected by NanoSight nanoparticle size analyzer (Malvern Panalytical NS300, UK).

### Animal model of MPS expansion and procedure

This study was approved by The China Medical University Ethics Review Committee (CMU2021480), following ARRIVE guidelines (Animal Research: Reporting of In Vivo Experiments) and the National Research Council's Guide for the Care and Use of Laboratory Animals. The sample size of experimental animals was derived following Arifin's Sample size formulas (degree of freedom (DF) = 4, N = 6) for group comparison [24]. Twenty-four male Sprague Dawley rats (6–7 weeks, 190 ± 10 g) were randomly assigned to four groups: the control (C) group (n = 6 rats), expansion-only (E) group (n = 6 rats), the M1 sEVs-assisted expansion (E + M1) group (n = 6 rats) and the M2 sEVs-assisted expansion (E + M2) group (n = 6 rats). For the C group, the expander was bonded without delivering expanding force while the latter three required wearing a personalized expander to receive the expansion treatment. The rats were kept in a clean-grade animal room with a 12 h-12 h light/dark cycle, controlled temperature (22–26℃), and humidity (55%-60%) at the Department of Laboratory Animal Science, China Medical University. The animals were provided with regular food and water ad libitum for 3 days before the experimental procedures. The operation of the MPS expansion model on rats was adapted from a previous study [25].

Before making the personalized expander, rats were anesthetized with 3% Sodium Pentobarbital (40 mg/kg) intraperitoneally on a rat dissection table to immobilize their limbs. The body temperature was maintained with a heated blanket throughout the experiment. The Imprint II Vinyl Polysiloxane Impression Material (3 M Uniek, USA) was used to take the maxillary impression, and then maxillary casts were made. The two-helical expansion springs device was made of 0.016-inch nickel-titanium wire (Forestadent, Germany) with arms symmetrical and covered the first and second molars at least. The expansion force of 70 ± 5 g was measured using an orthodontic strain gauge (Tiantian, China) when the two arms were compressed to contact the palatal side of the rat's molar area. After acid etching, flushing, and air drying, the expanders were bonded to the molars using light-curing composite resin (3 M Unitek, Z350XT 7018, Monrovia, Calif). The surfaces were smoothed to avoid occlusal traumatism. The unforced two-helical expansion springs were solely attached to the molar surface in the control group. In the E + M1 and E + M2 groups, 50µL M1 or M2 sEVs were injected submucosal at the midline between the palatine sutures of the first and second molar under isoflurane anesthesia daily so that 50 µg of sEVs were applied locally on each rat. Rats in group E received the same volume of 0.9 percent saline injection. All injections were performed with insulin syringes. The expansion duration was set at 7 days, soft food was provided with body weight and health was checked daily. Samples with severe mucosal injury or rapid weight loss were removed. Expanders were reinstalled immediately if separated. All rats were sacrificed by CO_2_ asphyxiation on the 7th day of expansion.

### Three-dimensional (3D) micro-computed tomography (μCT) analysis

The sacrificed rats' maxillary bones were harvested, immersed in 4% paraformaldehyde solution with the MPS expansion device, and fixed for more than 24 h at 4 °C. The expander was withdrawn after fixation, and a new maxillary impression was obtained to record the final state of expansion. Then the maxillas were analyzed using microcomputed tomography (µ‐CT) imaging system (SkyScan1276, Germany) from the incisor to the third molar region under 85 kV, 200μA with a resolution of 20 μm. We applied NRecon software (version 1.7.1.6, Bruker) to reconstruct 3D image datasets from 2D X-ray pictures. CT-AN software (version 1.17.9.0, Bruker) was used for tomographic image processing, and CTvox software (version 3.3.0.0, Bruker) was employed for 3D reconstruction. The linear distance between the bilateral bony edges within the first and second molars was measured on the coronal plane as the palatal suture width. To evaluate differences in bone volume (BV), the ratio of bone volume to tissue volume (BV/TV), and trabecular separateness (Tb. Sp) among groups, the maxilla was reoriented parallel to the axial plane using Dataviewer software (Version 1.5.4.6, Bruker). The area of interest (ROI) was defined as an area of 2 × 0.8 × 1 mm^3^ from the distal-palatal root of the first molar to the mesial-palatal root of the second molar and extended 400 μm from the bony edges bilaterally [5].

### Histochemical staining

The maxilla specimens were decalcified in 10% tetrasodium ethylenediaminetetraacetic acid (EDTA) solution for 4 weeks at 4 °C after the Micro CT scan. The samples were trimmed, dehydrated in graded ethanol, cleared with xylene, and embedded in paraffin with the palatal plane facing down. Longitudinal 5-μm-thick serial sections were taken in the axial plane and dried at 60 °C. Before staining, sections were deparaffinized and rehydrated in graded alcohol. Hematoxylin–eosin (HE) staining provides the morphological alterations of the midpalatal suture. For immunohistochemical staining, hydrated slices were blocked in 3% hydrogen peroxide solution for 30 min to inhibit endogenous peroxidase, then drop goat serum was for 30 min to block unspecific background binding. Each group's sections were chosen at random and incubated with rabbit anti-rat primary antibody (1:500, Abclonal, China), rabbit anti-rat P65 primary antibody (1:500, Abclonal, China), and rabbit anti-rat BMP-2 primary antibody (1:500, Abclonal, China) overnight at 4 °C in a humidity chamber. Subsequently, the sections were incubated with horseradish peroxidase (HRP) goat anti-rabbit IgG secondary antibody (Zhongshan, China) for 25 min at 37 °C. Sections were stained for 5–10 min with high-quality diaminobenzidine (DAB) substrate. The number of osteoclasts in the midpalatal suture was determined with a Tartrate-resistant acid phosphatase (TRAP) staining kit (387A-1KT, Sigma, USA) following the manufacturer’s instructions. Hematoxylin was used to counterstain the nuclei.

A light microscope (DM4000B, Leica, Germany) was used to examine the stained sections, and microphotographs were taken by an electronic camera system (V4.12, LAS). The region of interest for RANKL and P65 expression and osteoclast were near the bone margin while the area of interest for BMP-2 expression was in the middle of the palatal suture, according to previous research [5, 26]. Cells with brownish-yellow granules in the cytoplasm or nucleus were considered to be positive cell characteristics. Osteoclasts were defined as multinucleated cells on the surface of the bone. Image-Pro Plus software (version 6.0; Media Cybernetics, Bethesda, Md) was used to calculate the mean optical density (MOD) of immunohistochemistry images. For each slice, a single examiner evaluated three fields of view at random, and the results were averaged across the three fields.

### Statistical analysis

The data were supplied in the format of mean ± standard deviation (SD). The IBM SPSS software (version 22.0, USA) was used for statistical analysis and data processing. The Kolmogorov–Smirnov test was performed to check the normality of the data. For the comparison of body weight, the independent samples t-test was utilized. Comparison between groups was conducted using one-way analysis of variance (one-way ANOVA) with Fisher's Least Significance Difference post-hoc (LSD) test. All tests were two-sided. Significant differences were defined as p < 0.05 at most (*P < 0.05, **P < 0.01, ***P < 0.001).

## Results

### Identification of the M1, M2 macrophage phenotypes

**Fig. 1:**
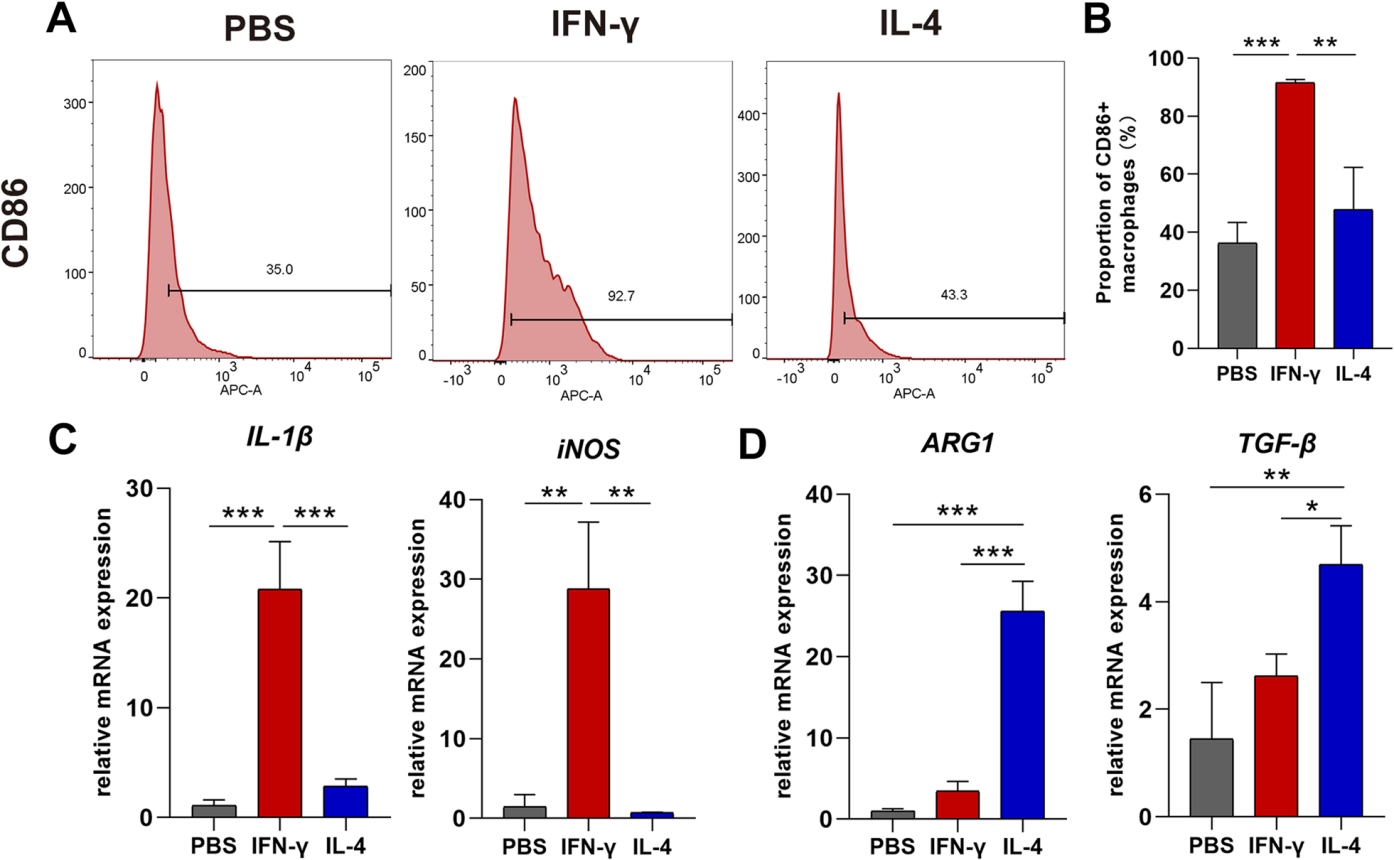
Identification of M1, M2 polarized macrophages following PBS (control), IFN-γ or IL-4 stimulus. **A** Expression of CD86 (M1 marker) in polarized RAW 264.7 cells analysed by flow cytometry; **B** Statistical results of the proportion of polarized RAW 264.7 cells from flow cytometry; **C**–**D** Gene expression in polarized RAW 264.7 cells analysed by qRT-PCR. IL-1β, INOS were used as M1 markers; ARG1, TGF-β were used as M2 markers; normalized to GAPDH and relative to PBS group (unstimulated cells). Data were collected from three independent experiments and presented as mean ± SD. Differences were analyzed by one-way ANOVA (**B**–**D**. *P < 0.05, **P < 0.01 and ***P < 0.001 represent significant differences between the groups. Arg1, Arginine-1; IFN-γ, interferon γ; IL-1β, Interleukin-1 β; IL-4, interleukin-4; iNOS, inducible nitric oxide synthase; mRNA, messenger RNA; SD, standard deviation; TGF-β, Transforming growth factor-β; TNF-α, tumor necrosis factor-α

For the stimulation of M1 and M2 macrophages, we used a cytokine-induced protocol, following flow cytometry and RT-PCR for cell subtype identification. CD86 is one of the most common M1 macrophage surface markers. In comparison to PBS-treated and IL-4-treated M0 macrophages, IFN-γ-treated M0 macrophages had a considerable upregulation of CD86-positive rate (Fig. 1A). The CD86-positive macrophages in the IFN-γ-treated group were considerably greater than those in the IL-4-treated and PBS-treated group (Fig. 1B; P < 0.01 or P < 0.001). Quantitative real-time PCR was used to identify different macrophage surface marker mRNA expression levels. The results revealed that macrophages treated with IFN-γ had significantly higher levels of iNOS and IL-1 expression than the other two groups (Fig. 1C, P < 0.01 or P < 0.001), while IL-4-treated M0 macrophages expressed higher levels of M2 macrophage markers TGF-β and ARG 1 (Fig. 1D, P < 0.05 or P < 0.001). These results demonstrate that cytokine (IFN-γ and IL-4) successfully stimulate macrophages into M1 and M2 phenotypes.

### Characterization of M1, M2 macrophage sEVs

**Fig. 2:**
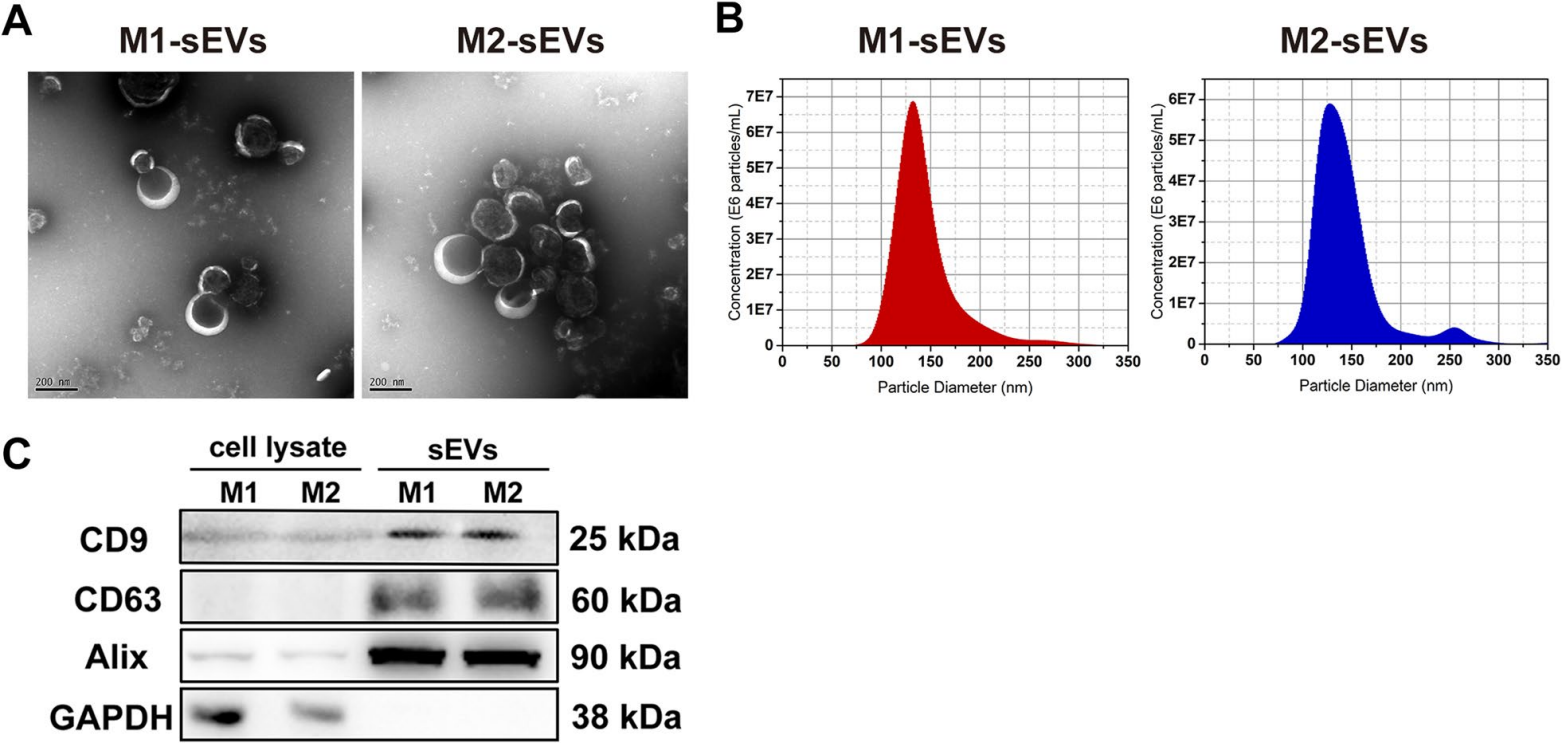
Characterization of sEVs derived from M1, M2 polarized macrophages. **A** Representative TEM images of the M1-sEVs and M2-sEVs. The structure of M1, M2-sEVs were revealed; **B** Nanoparticle tracking analysis of the M1-sEVs and M2-sEVs. The size distribution of M1, M2-sEVs were presented, respectively; **C** The presence of EVs marker proteins (CD9, CD63, Alix) in M1, M2 cell lysate and M1, M2-sEVs (Western blot analysis). sEVs, small-sized EVs; TEM, transmission electron microscope

We isolated and analyzed sEVs produced by M1 and M2 macrophages, respectively. The M1 and M2-sEVs had bilayer membrane structures and cup-shaped morphology. There was no significant difference between the two groups (Fig. 2A). The size distribution of sEVs revealed a concentrated tendency in nanoparticle tracking analysis (NTA). M1 macrophage-sEVs had an average particle diameter of 143.6 ± 32.7 nm at a concentration of 3.08 × 10^11^ particles/ml, while M2 macrophage-sEVs had an average particle diameter of 143.6 ± 35.2 nm at a concentration of 6.76 × 10^11^ particles/ml. The particle diameter of the two was consistent with TEM (Fig. 2B). Western blot analysis demonstrated the expression of membrane protein markers CD9, CD63, and Alix in sEVs (Fig. 2C). The internal control GAPDH was exclusively found in cell lysates. Therefore, following the guidelines [14] the isolated vesicles can be classified as small-sized EVs (sEVs). For animal investigations, protein concentration was measured with BCA kit to indicate the number of sEVs.

### M1 macrophage sEVs enhance MPS expansion in vivo

**Fig. 3:**
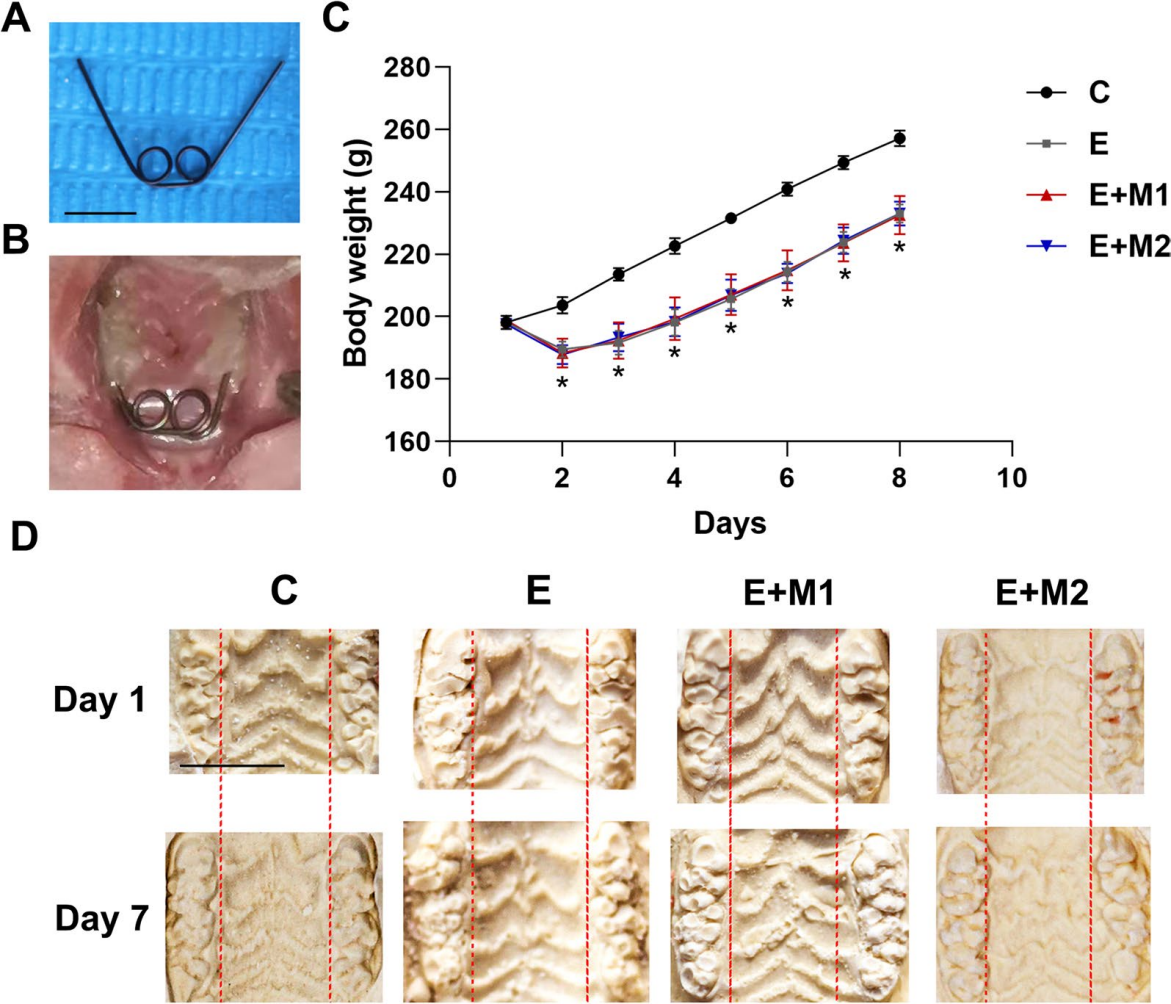
Body weight statistics and analysis of model photos after arch expansion. **A** Occlusal view of the suture expander; **B** Intra-oral view of the suture expander; **C** Changes of body weight during expansion; *P < 0.05, significant decrease versus the control group; **D** Photos of rat palate model before and after suture expansion. The buccal inclination of the molars can be noted in all groups, with the E + M1 group manifested the most evident effect. C, control group; E, expansion-only group; E + M1, M1 sEVs-assisted expansion group; E + M2, M2 sEVs-assisted expansion group. Scale bar: 5 mm

Following the commonly documented method, we bonded the bilateral posterior teeth together to reflect the bony effect of the expansion force (Fig. 3A&B) [3, 25]. Expanders are adapted well in samples and no trauma or inflammation was witnessed. One rat died during the experiment, and another slipped off the expander for more than 24 h, with prompt supplementation carried out. We locally infused saline and M1/M2-sEVs into E, E + M1, and E + M2 groups in the midpalatal suture and analyzed the effects at 7 days. The weight growth of the C group rose at a rate of roughly 7–10 g/day while the other three groups dropped weight sharply at first, then gradually reached the rate of the C group by the fourth day. After acclimatization, all rats were able to access soft food and sustain a consistent weight gain. The bodyweight of the expansion-treated group was consistently significantly lower than that of the control group throughout the experiment (P < 0.05, Fig. 3C). After 7 days, the palatal folds from all rats tended to develop larger and wider. Group E, E + M1, and E + M2 exhibited larger widths between molars and evident molar tipping than the baseline condition, and the palatal vault became shallow and flat (Fig. 3D), especially in group E + M1. This suggests that the expander has a dentoalveolar effect, and M1-sEVs injection can help improve expansion effectiveness to some extent.

### M1 macrophage sEVs promote MPS width

**Fig. 4:**
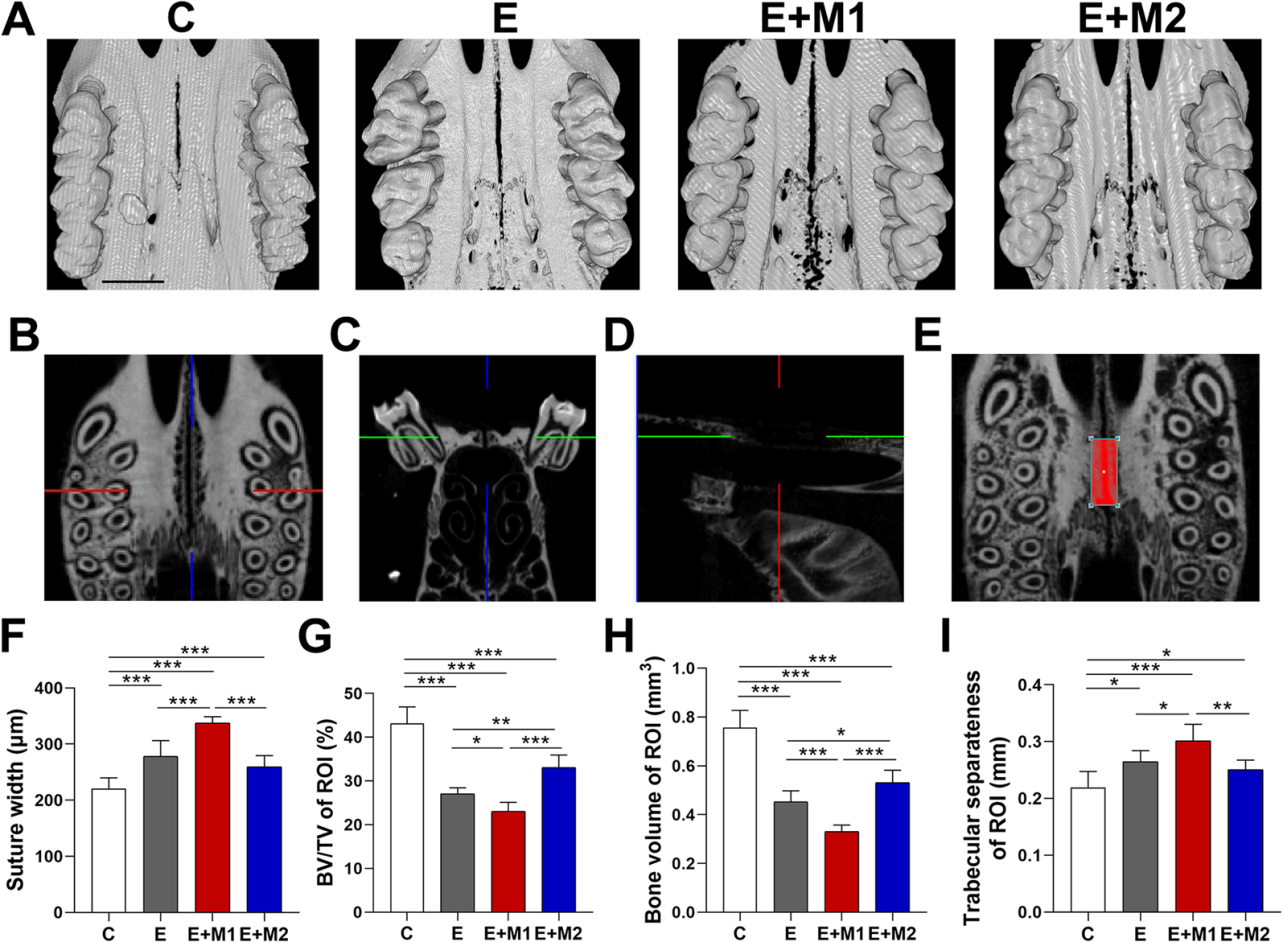
Three-dimensional (3D) micro-computed tomography (μCT) analysis. **A** Representative images of three-dimensional Micro CT reconstruction of MPS; **B** Axial images of MPS; **C** Coronal images of MPS; **D** Sagittal images of MPS; **E** The region of interest. BV, BV/TV and Tb. Sp were measured within ROI (2 mm × 0.8 mm × 1 mm) from the distal-palatal root of the first molar to the mesial-palatal root of the second molar and extended 400 μm from the bony edges bilaterally; Data were presented as mean ± SD. Differences were analyzed by one-way ANOVA (**F**–**I**). Comparative analyses of the Micro CT measurements on **F** suture width; **G** bone volume to tissue volume (BV/TV); **H** bone volume (BV) and **I** trabecular separateness (Tb. Sp). (* P < 0.05, ** P < 0.01, *** P < 0.001). Scale bar: 2 mm

The specimens were scanned by Micro CT to evaluate changes in the midpalatal suture and the bone structure in the ROI to determine the effects of M1/M2-sEVs. The significant difference in the midpalatal suture could be observed in 3D model reconstruction (Fig. 4A). Orient the maxilla correctly to get the most consistent ROI (Fig. 4B–E). The suture width was substantially broader in groups E (278.26 ± 27.8 μm), E + M1 (337.49 ± 11.2 μm), and E + M2 (273.96 ± 47.11 μm) than in group C (220.47 ± 19.22 μm) (P < 0.001), in which E + M1 group was significantly greater than any other group (P < 0.001). However, E + M2 was narrower than E with no statistically significant difference (P > 0.05) (Fig. 4F).

According to volumetric analysis, the expansion samples showed a significant decrease in BV/TV than the C group (P < 0.001). The BV/TV of the E + M1 group (23.03 ± 2.07%) was considerably lower than that of the E group (27.10 ± 1.34%) and the E + M2 group (33.59 ± 8.13%) (P < 0.05, P < 0.001). In addition, BV/TV in the E + M2 group was significantly greater than in the E group(P < 0.01) (Fig. 4G).

Likewise, bone volume presented a trend consistent with BV/TV (Fig. 4H). Significant differences were observed among the E + M1 group (0.32 ± 0.03 mm^3^) with C group (0.76 ± 0.07 mm^3^), E group (0.45 ± 0.05 mm^3^), and E + M2 group (0.53 ± 0.05 mm^3^) (P < 0.001).

Trabecular separateness in the E + M1 group (0.30 ± 0.03 mm) was significantly higher than in the E group (0.26 ± 0.02 mm) and E + M2 group (0.26 ± 0.04 mm) (P < 0.05, P < 0.01). No difference was witnessed between the E + M2 group and the E group (P > 0.05) (Fig. 4I).

### Morphology changes during MPS expansion

**Fig. 5:**
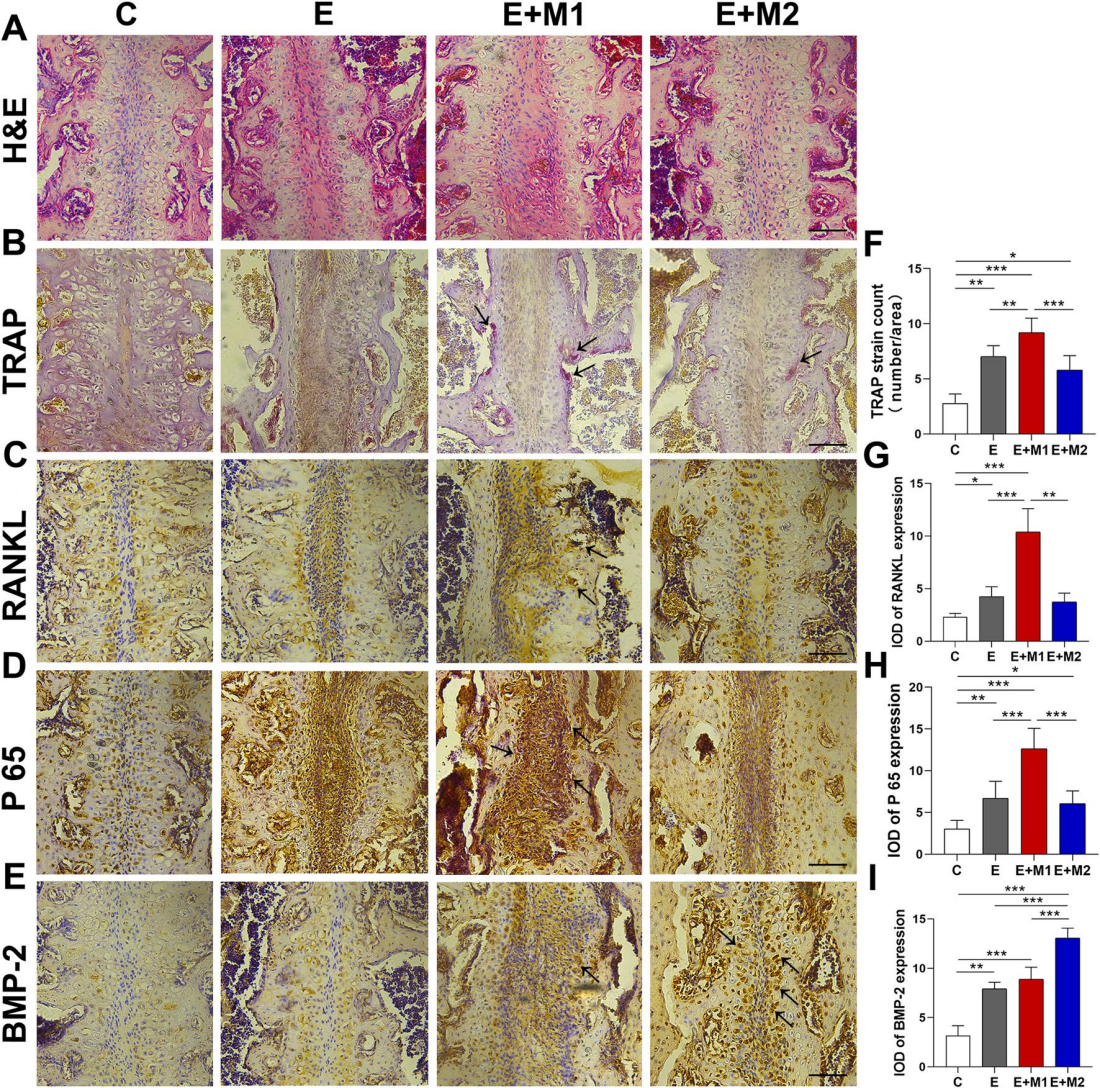
Immunohistochemical staining of midpalatal suture of different groups. **A** Hematoxylin and eosin staining (**H**&**E** staining) of midpalatal sutures after expansion; **B** Identification of osteoclasts (TRAP staining); **C** Expression of RANKL; **D** Expression of p 65; **E** Expression of BMP-2. **F** Tartrate-resistant acid phosphatase (TRAP)-positive count; **G**–**I** Semiquantitative analysis of RANKL, P 65 and BMP-2; Data were presented as mean ± SD. Differences were analyzed by one-way ANOVA (**F**–**I**). Black arrows point to the TRAP-positive osteoclast (**B**), RANKL positive tissue (**C**), p 65 positive tissue (**D**) and BMP-2 positive tissue (**E**). The E + M1 group displayed significantly higher levels of RANKL and P65 protein expression than any other group and the osteoclast counts were significantly greater. The E + M2 group demonstrated an increase in BMP-2 expression (* P < 0.05, ** P < 0.01, *** P < 0.001). Scale bar: 100 μm

We utilized HE staining to reveal the histological manifestations of each group (Fig. 5A). The palatal suture in the control group included thin layers of fibrous tissue in the center and cartilage tissue on both sides that contained chondrocytes. In the E group, the fibers between the palatine bones were altered and the cartilage tissue was separated slightly. Expansion had a noticeable impact on the E + M1 group, as evidenced by abundant fiber rearrangements in the transverse direction parallel to the direction of MPS expansion and an increase in the space between the palatine bones. In comparison to the E + M1 group, the E + M2 group's performance on expansion was insignificant.

### Effect of M1 macrophage sEVs during MPS expansion

We used TRAP staining to count the osteoclasts in the palatal area. In the E, E + M1, and E + M2 groups osteoclasts were significantly increased along the lateral edge of the bone compared to the control group (Fig. 5B, F). The lowest number of osteoclasts, a physiological condition, was seen in the control group. Osteoclast counts in the E + M2 group were less than in the E group (P > 0.05). There were a considerable number of TRAP-positive cells that appeared in the E + M1 group (P < 0.01). The integrated optical density analysis of RANKL produced similar findings (Fig. 5C, G). The E + M1 group displayed significantly higher levels of RANKL and P65 protein expression than any other group (P < 0.01), with the main expression located at the lateral border of the bone. A significantly greater distance between the palatine bones was observed in the E + M1 group. The three groups with MPS expansion expressed higher P65 protein expression, with the E + M1 group having the greatest expression (P < 0.001), while there was no significant difference between the E group and the E + M2 group (Fig. 5D, H). Additionally, the E + M2 group demonstrated a substantial increase in BMP-2 expression in the central region of the palatal suture (P < 0.001) in comparison to the other three groups, while the E and E + M1 groups both were greater than the control group(P < 0.01) (Fig. 5E, I).

## Discussion

A multitude of factors should be considered for MPS expansion treatment to be successful. There are currently few pharmacological means or carrier-packaged drugs to modulate suture remodeling [19], such as matrine [29], parathyroid hormone [5], and salvianolic acid B [30]. However, researchers have primarily concentrated on promoting the osteogenesis of the MPS after expansion to reduce recurrence. In this study, we provided evidence that M1-sEVs promote MPS expansion. A suspension of M1/M2-sEVs was administered locally to the palatal suture of rats at a dose of 50 µg/day. Compared with those of the E and E + M2 groups, both BV/TV and bone volume were decreased in the E + M1 group (Fig. 4G–I). HE staining indicated that M1-sEVs promoted palatal suture expansion (Fig. 5A). This result proves that the E + M1 group exhibited the largest palatal suture, and the proportion of bone mass in the ROI decreased. Immunohistochemical (IHC) analysis of MPS expansion showed that the expression of RANKL and P65 protein at the edge of the palatine bone in the E + M1 group was significantly enhanced, and the number of osteoclasts increased (Fig. 5F–H). By affecting p65 expression and activating the NF-κB pathway, M1 macrophage sEVs enhanced the inflammatory process and receptor activator of NF-κB ligand (RANKL) expression in the early stage of bone turnover, exhibiting a more noteworthy influence on palatal tissue rearrangement. As a cell-free therapy, topical application of sEVs is less traumatic, more stable and readily absorbed, and shows better promotion of local drug repurposing than systemic application [31]. These properties may contribute to the development of unique drugs containing M1-sEVs to safely and effectively enhance the clinical therapeutic effect of RME, and EV-related signaling molecules may be promising targets for new approaches.

The structure of the palatal suture in the posterior region of rats is similar to that of humans, with secondary cartilage along the palatine bone edges and a thin layer of fibers in the core, with suture-derived stem cells (SuSCs) in the center [3, 32]. SuSCs are a unique functional population that possess an intrinsic ability to differentiate into osteoblasts, chondrocytes, and fibroblasts[33]. The mechanical stretching force of suture expansion causes macrophages infiltrate into the midpalatal suture within 24 h [6]. Studies have shown that to promote bone formation, macrophages recruited by stress in the palatal suture are often polarized into M2 macrophages, thereby stimulating osteogenic differentiation [9]. Additionally, T-cell subsets such as Th1 cells and Th17 cells have been shown to infiltrate into expansion sutures [34]. The recruitment of these immune cells can activate the immune response and promote SuSC proliferation and osteoblast differentiation. In fact, M1 and M2 macrophages have opposite effects on bone repair [7]. The role played by M1 macrophages in the expansion of the midpalatal suture remains unclear. This study demonstrates that the initial phase of the inflammatory process is critical for the expansion of the midpalatal suture by local injection of M1-sEVs. Further studies are needed to determine the role and mechanism of M1 macrophages and M1-sEVs in bone remodeling in the expanded midpalatal suture.

A moderate 70 g force was chosen according to the literature to maintain expansion results and avoid recurrence [30]. None of the animals showed negative effects such as weight loss. Rats continued to gain weight after being accustomed to the expander, and all groups displayed a widening of the suture fold after expansion (Fig. 3D). The palatine bone still significantly widened in the E + M1 group (Fig. 3D), demonstrating the effectiveness of sEV therapy conducted in this study. The 7-day expansion duration in rats is almost equivalent to the 6-month expansion treatment time in humans [5, 36], which is also the typical amount of time needed for MPS therapy in clinical training. Approximately 0.3 mg EV protein/kg body weight has been determined to be the therapeutic dose for rats in dose-related investigations of the in vivo test [37]. At the beginning of the trial, the rats' weights were less than 200 g, and the recommended dose was calculated to be approximately 60 µg. Previous studies employed daily intermittent subcutaneous injections of parathyroid hormone to evaluate its impact on the outcomes of arch expansion [5]. Finally, we administered 50 µg/day injections to ensure that the EVs continued to function during expansion.

A previous study showed that LPS-treated macrophages could activate TNF/NF-κB signaling and P-p65 expression and establish a local inflammatory environment [16, 38, 39]. Similarly, M1-polarized macrophage EVs upregulated miR-155 with inhibited SOCS6-induced p65 degradation and regulated the NF-κB pathway to induce inflammation in a spinal cord injury study [40]. NF-κB is responsible for the transcription of many genes coding proinflammatory cytokines and chemokines. Nuclear translocation of p65, an indicator of activation of the NF-κB p65 signaling pathway, may bind to the promoters of inflammatory genes and then regulate inflammation [41]. Local sEV injection may have an immediate impact on cells and control signal transduction via the paracrine pathway [42]. This study showed that the E + M1 group had significantly enhanced expression of p65, suggesting that M1-sEVs could significantly improve the activity of NF-κB by increasing the content of p65 protein, showing a proinflammatory state. The section staining indicates that the p65-positive cells are located close to the palatal bone margin and are closely associated with the expression of osteoclasts. However, since we analyzed the optical density of the entire section, this finding may not suggest the precise location of the p65-positive cells.

The binding of RANKL to the receptor activator of nuclear factor-κB (RANK) on the surface of osteoclast precursor cells not only promotes osteoclast activation but also serves as an initiating factor of NF-κB [43]. Yi [5] demonstrated that continuous parathyroid hormone increased the expression of RANKL, increased the number of osteoclasts near the lateral bone edge, and significantly promoted the expansion impact, which is consistent with our results. At the beginning of MPS expansion, soluble RANKL is elevated in rat serum, which may be the result of tissue necrosis and subsequent proteolytic or alternative mRNA splicing of transmembrane receptors [44]. The contact of the EV contents with palatal tissue cells by injection of M1-sEVs also contributed to the enhanced expression of RANKL, which promoted the differentiation of osteoclast precursors to osteoclasts, leading to the start of bone remodeling [45].

**Fig. 6:**
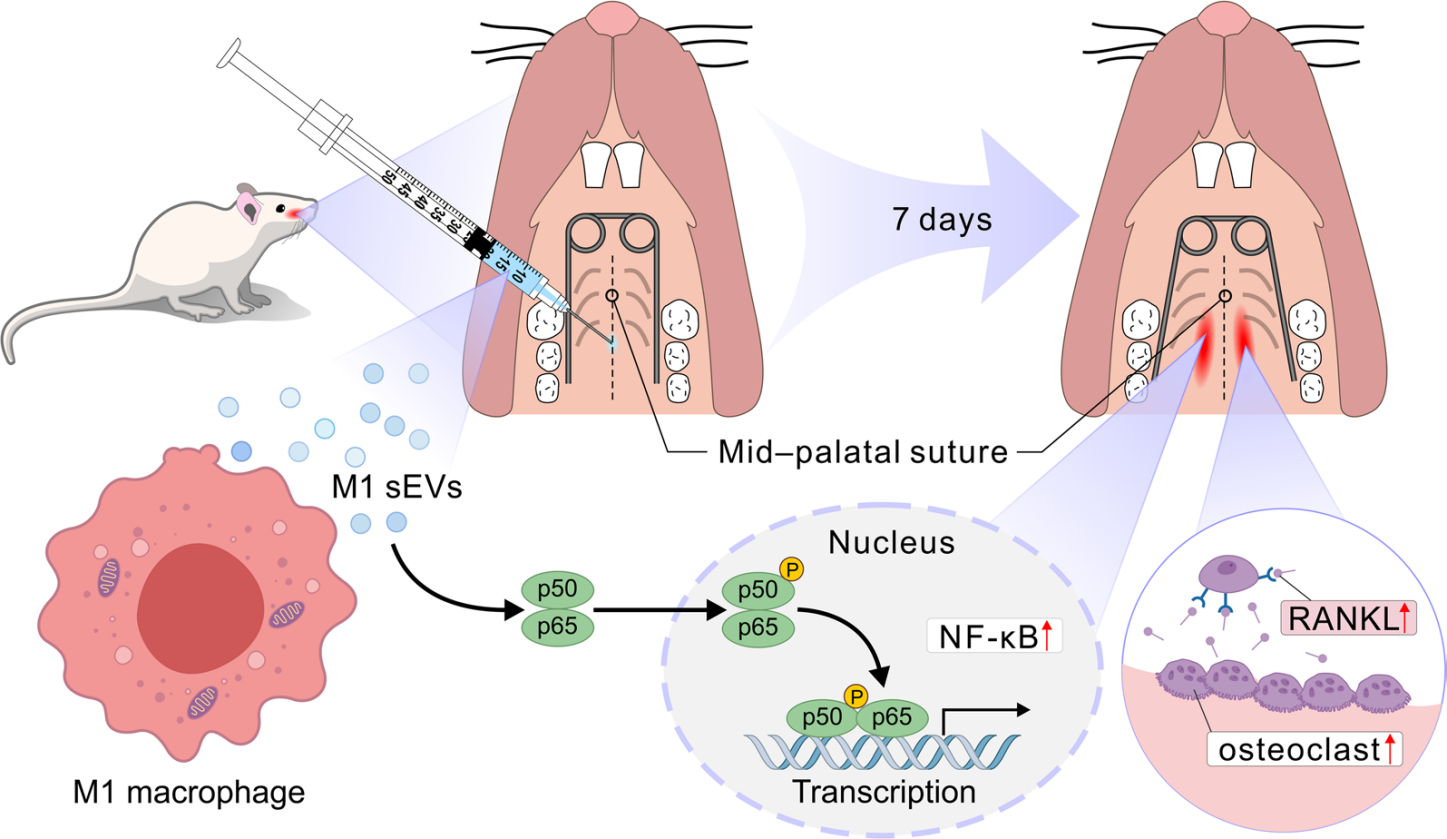
Schematic illustration of the mechanism by which M1 sEVs enhance palatal suture expansion. M1 sEVs promote the early inflammatory state by increasing the content of RANKL and promoting the formation of osteoclasts, thus increasing the effect of bone turnover. They also promote the activation of the NF-κB signaling pathway in the palatal region by increasing the content of p65 protein

Similar to the local area acceleration phenomenon (RAP) of rapid orthodontic tooth movement, the promotion of osteoclast bone resorption and concomitant coupling of bone remodeling during inflammation is a potential mechanism for rapid expansion [36]. Previous findings suggested that when osteoclasts and bone turnover were inhibited, expansion was attenuated and fibers dispersed, confirming a key role of inflammation in the expansion of the palatal suture [4]. A transient and controlled increase in the inflammatory state or elevated numbers of osteoclasts promotes faster bone remodeling, i.e., increased bone turnover [50]. After "prolonging" or "promoting" early inflammation, subsequent cytokines and growth factors can activate new bone formation. Osteoclast activation should theoretically be accompanied by greater bone resorption and bone formation, but reductions in bone fraction (BV/TV) and bone volume (BV) were observed, suggesting that bone resorption outpaced bone formation during our 7-day treatment period. This finding is consistent with Jacobx [51], who showed that the inflammation-related expression and bone development seemed to progress in an orderly manner. Inflammation, autophagy, and bone breakdown predominate after the force is applied, but bone formation overtakes inflammation and subsides by Day 7. From the results of immunohistochemistry and the physiological state of animals, the increase in inflammation caused by M1-sEVs did not cause tissue tearing or biological function decline, but the controllability of inflammation requires further investigation. In Fig. 6, we outline the present mechanism by which M1 sEVs encourage palatal suture expansion.

BMP-2 mainly acts as a differentiation factor of bone and cartilage precursor cells and can promote bone regeneration and remodeling during bone repair. Kang [18] found that BMP signaling was significantly increased after M2-EV treatment of mouse calvarial defects. We used M2-sEVs as a control group to investigate the effect on MPS expansion as M1 and M2 are two subtypes of polarized macrophages. From the in vivo experiments and micro-CT results, we found that although the E + M2 group had a significantly thinner palatal suture width than the E + M1 group, it was not significantly different from the E group (Fig. 4A&F). However, the bone volume and BT/TV were significantly larger than those in the E group. Immunohistochemical results showed increased BMP2 expression in the E + M2 group compared to all the other groups (Fig. 5E, I). In contrast, both the E and E + M1 groups showed a relative decrease in BMP2 expression. M2-sEVs may produce osteoprotective effects that promote osteogenesis and inhibit osteoclastogenesis by delivering IL-10 mRNA or delivering miR-27a-3p [52]. In this study, the E + M2 group did not show a completely positive effect in promoting bone formation in the expanded palatal suture region of the MPS (relative to the expansion-only group), but Xia confirmed that M2-sEVs mainly act in the late stage of osteogenic differentiation [23]. There is reason to suspect that the less pronounced effect of M2-sEVs is related to the shorter duration of expansion.

Our research has several limitations. First, further studies are needed to identify EV-targeted tissues and to explore the effect of M1-sEVs on SuSC differentiation in the palatal suture. Second, we did not explore bone regeneration during the maintenance phase of the palatal suture after expansion or stability, which may be clarified in further studies. Third, adult individuals should be used in the future to study the effect of M1-sEV on bony MPS expansion. Future studies should focus on identifying the mechanisms of inflammatory signaling in M1-sEVs and exploring the relevant mRNA targets in the mechanisms.

## Conclusion

The M1 macrophage-derived sEVs were a unique and effective method for palatal suture expansion. Our data showed that M1-sEVs enhanced midpalatal suture expansion by promoting initial inflammation, RANKL expression, and osteoclastogenesis in the MPS, partially by regulating the NF-kB p65 pathway.

## Data Availability

The datasets used and/or analysed during the current study are available from the corresponding authors on reasonable request.

## Abbreviations

MPS: Midpalatal suture
EVs: Extracellular Vesicles
sEVs: Small-sized EVs
DMEM: Dulbecco Modified Eagle Medium
PBS: Phosphate buffer saline
FBS: Fetal bovine serum
BSA: Bovine serum albumin
IFN-γ: Interferon-γ
IL-4: Interleukin-4
Arg-1: Arginase-1
iNOS: Inducible nitric oxide synthase
IL-1β: Interleukin-1β
TGF-β: Transforming growth factor-β
NF-κB: Nuclear factor-kappa B
RANKL: Receptor activator of nuclear factor kappa-B ligand
RANK: Receptor activator of nuclear factor-κB
HE: Hematoxylin and Eosin
TRAP: Tartrate-resistant acid phosphatase
MOD: Mean optical density
SuSCs: Suture-derived stem cells
